# Generalized structural equation modeling of intimate partner violence among married women in East Africa using population-based data

**DOI:** 10.1038/s41598-026-44933-3

**Published:** 2026-03-18

**Authors:** Bewuketu Terefe, Fentahun Bikale Kebede, Debela Nanesso Gatira, Solomon Keflie Assefa, Yalelet Fentaw Shiferaw, Nega Tezera Assimamaw, Tsegereda Abebe Andargie, Dejen Kahsay Asgedom

**Affiliations:** 1 School of Nursing, College of Medicine and Health Sciences, University of Gondar, 196, Gondar, Ethiopia; 2 Stratigic Affairs Executive Office, Ministry of Health, Addis Ababa, Ethiopia; 3 Department of Emergency and Critical Care Nursing, School of Nursing, College of Medicine and Health Sciences, University of Gondar, Gondar, Ethiopia; 4 Pawe Health Science College, Northwest, Ethiopia; 5 Department of Epidemiology, and Biostatistics, Institute of Public Health, College of Medicine and Health Sciences, University of Gondar, Gondar, Ethiopia; 6 6Department of Nutritional Care and Counseling, University of Gondar Specialized Hospital, Gondar, Ethiopia; 7 Department of Pediatrics and Child Health Nursing, School of Nursing, College of Medicine and Health Sciences, University of Gondar, Gondar, Ethiopia; 8 International Institute for Primary Health Care-Ethiopia, Addis Ababa, Ethiopia; 9 Department of Public Health, College of Medicine and Health Sciences, Samara University, Afar, Ethiopia

**Keywords:** Structural equation model, Intimate partner violence, Gender-based violence, Married women, East Africa, Health care, Medical research

## Abstract

Intimate Partner Violence (IPV) remains a persistent public health and social problem among women in Africa, with severe physical, emotional, and sexual consequences that hinder well-being and socio-economic development. Despite ongoing global and regional efforts, evidence on the prevalence and determinants of IPV across East African countries is limited, fragmented, and often focused on single countries. A more comprehensive, regional-level understanding is crucial to guide targeted interventions and policies This study aimed to provide a comprehensive assessment of IPV among married women in East Africa by (1) estimating the prevalence of physical, emotional, and sexual IPV across 12 countries, and (2) identifying individual-, household-, and community-level factors associated with IPV using a structural equation modeling (SEM) approach. By integrating multi-country data, the study seeks to generate region-wide evidence that can inform prevention strategies and policy responses to gender-based violence in East Africa. A nationwide community based cross-sectional study was conducted in 12 East African countries as part of the Demographic, and Health Survey (DHS) in the region. The study gathered data from 56,657 married women through structured questionnaires. Descriptive findings were presented as percentages with a 95% confidence interval. To identify factors associated with different types of IPV (physical, emotional, and sexual), a Generalized Structural EquationModel was used. Adjusted odds ratios (AOR) with a 95% confidence interval were used to determine the significant factors related to IPV. The pooled prevalence of overall IPV in East Africa countries was 38.03% (95% CI: 33.23, 43.08). The highest prevalence of IPV was found in Uganda (53.24%), whereas the lowest was in Comoros (10%). Older age, secondary/higher education, wealth, current working status, attitude towards wife beating, husband’s educational status, husband’s current working status, and husband’s drinking alcohol were factors associated with physical violence. Regarding emotional violence, older age, primary education, wealth, media exposure, current working status, smoking cigarettes, women’s decision-making autonomy, attitude towards wife beating, husband’s education, and husband’s drinking alcohol were significant factors. Finally, regarding sexual violence, factors such as rural residence, better education, wealth, working status, women’s decision-making autonomy, husband’s education, and husband’s drinking alcohol were associated with it. The findings indicate a high prevalence of IPV among married women in East Africa. Key factors for physical violence include age, education, economic status, and attitudes toward domestic violence, while emotional and sexual violence are linked to women’s decision-making autonomy and their partners’ behaviors. To address IPV, comprehensive education and awareness programs are essential, emphasizing gender equality and healthy relationships. Additionally, policies should focus on enhancing women’s economic empowerment and decision-making autonomy, supported by community initiatives to challenge harmful norms.

## Introduction

Violence is a major global public health concern with profound consequences for individuals, families, and communities. The World Health Organization (WHO) defines violence as the intentional use of physical force or power, whether threatened or actual, against oneself, another person, or a group or community, that results in or has a high likelihood of resulting in—injury, death, psychological harm, maldevelopment, or deprivation. This comprehensive definition highlights that violence extends beyond physical harm to include psychological and structural dimensions that affect health and well-being. A specific form of this, intimate partner violence (IPV), refers to behavior within an intimate relationship that causes physical, sexual, or psychological harm, including acts of physical aggression, sexual coercion, psychological abuse, and controlling behaviors. This definition encompasses violence perpetrated by both current and former spouses or partners, highlighting the multifaceted nature of violence in close relationships^1–3^. Intimate partner violence (IPV) is the most common form of violence against women^1^. According to the World Health Organization (WHO), approximately 27% of women aged 15–49 who have ever been married or in a partnership have experienced physical or sexual violence, or both, by an intimate partner at least once in their lifetime. The prevalence of IPV over 12 months is estimated to be 13%^1^. However, IPV is prevalent in developing countries, particularly in African countries where male dominance holds significant power.

Studies indicate that IPV poses a significant public health concern for women of reproductive age. Women who experience physical and sexual violence from their intimate partners face a 50–70% risk of developing gynecological, central nervous system, and stress-related problems^3^. Furthermore, multiple studies have argued that IPV (Intimate Partner Violence) also has mental and physical health consequences for women. Specifically, it has been found to lead to depressive symptoms and the loss of social and professional networks. This is often due to feelings of stigmatization and the need to be absent from work. As a result, women may also pRefer to be alone^4–6^. It is important to note that intimate partner violence is a significant public health issue in sub-Saharan Africa^7,8^.

Studies from several African countries show that intimate partner violence (IPV) is a significant public health concern for women, children, and fetuses, and it increases over time. This violence exposes them to various psychosocial health problems. For example, approximately 41% of ever-married women in Kenya and 21.5% of women in Nigeria of reproductive age have experienced IPV at some point in their lives^9,10^. Similarly, in Ethiopia, about 71% of women have been exposed to IPV, with emotional or psychological violence being the most prevalent (57.8%), followed by physical violence (32.2%) and sexual violence (7.6%)^11^. According to the latest Liberian Demographic and Health Survey report, the prevalence of IPV among ever-married women in Liberia has increased from 49% in 2013 to 60% in 2019/20^12^.

While a simple regression analysis may not provide a definitive explanation for the underlying causes, previous studies on IPV have identified several factors that are significantly associated with a decreased or increased likelihood of experiencing intimate partner violence. These factors include older age, higher educational status of women, urban residence, having an educated husband, and exposure to mass media. Conversely, factors such as alcohol abuse by the partner, women’s positive attitude towards wife beating, and polygynous marriage are significantly associated with an increased likelihood of being exposed to intimate partner violence^6,11,13–16^.

Studying IPV through the use of structural equation model (SEM) can provide valuable insights into the complex dynamics and underlying factors associated with IPV in this specific population^17,18^. IPV is influenced by various individual, relational, community, and societal factors, making it a complex issue. By using SEM, researchers can examine the interrelationships between these factors and assess their direct and indirect effects on IPV. Additionally, SEM allows for the inclusion of latent constructs, which are unobserved variables that cannot be directly measured. These latent constructs can represent underlying factors such as power dynamics, gender norms, or psychological well-being in the context of studying IPV^17–19^. By incorporating latent constructs in the model, policymakers can better understand the underlying factors contributing to IPV among rural women.

To the best of our knowledge, there remain population-level, methodological, and evidence gaps regarding intimate partner violence (IPV) in East Africa. Although a few studies have explored IPV, they have generally not been conducted across multiple East African countries, nor have they examined the full range of associated factors across different IPV domains. Importantly, most prior studies relied on traditional approaches such as logistic regression or multilevel modeling. While valuable, these models are limited in that they treat each IPV domain (physical, emotional, sexual) as separate, independent outcomes and cannot adequately account for the interrelationships among them or the latent constructs underlying IPV risk factors, such as husband controlling behavior.

By contrast, the Generalized Structural EquationModel (GSEM) provides methodological advantages that make it particularly well-suited for this study. GSEM allows the simultaneous analysis of multiple outcome variables within a single framework, enabling us to capture the three domains of IPV as related but distinct phenomena. It also permits the inclusion of latent constructs measured by categorical indicators, which is critical in IPV research where many key factors (e.g., controlling behaviors, psychosocial dimensions) are not directly observed. Moreover, GSEM extends beyond the assumptions of logistic and multilevel models by accommodating categorical outcomes, correlated error structures, and complex pathways linking predictors to multiple IPV domains.

Therefore, in this study we applied GSEM to investigate both the prevalence of IPV and its determinants using recent national health survey data from East Africa. This approach not only addresses methodological gaps left by previous studies but also generates more nuanced insights into the interconnected nature of IPV, offering evidence that is both epidemiologically robust and practically relevant for healthcare providers, program developers, and policymakers.

## Methods

### Study setting, period, and time frame

The data were obtained from the most recent standard DHS dataset of East African countries (2011–2022) (Table 1). A standardized dataset was used^20^ to obtain all parameters and a large sample size that is representative of the population source. DHS collects data that is cross-nationally comparable. The surveys are population-based and nationally representative of each country, with large sample sizes^20^. Eastern Africa consists of 14 countries situated in the Great Lakes region, the Horn of Africa, and the Indian Ocean Islands. These countries share common economic, social, and environmental challenges and are worried about their ability to achieve all of the targets set by the Millennium Development Goals^21^. East Africa is a region in the eastern part of the African continent, which includes the Saharan Desert and areas in the Horn of Africa. According to the United Nations, it spans an area of 6,667,493 square kilometers (2,574,332 square miles) and is home to approximately 6.03% of the world’s population, with a total of 491,899,592 people^22^.

**Table 1:** Country, years of survey, and ample size of Intimate Partner Violence (IPV) in East African countries.

East African countries	DHS year	Sample	
		Unweighted	Weighted
Burundi	2016/17	6,401	6611
Ethiopia	2016	4,123	4422
Kenya	2022	10,886	10,625
Comoros	2012	2,302	2334
Madagascar	2021	4,991	4994
Malawi	2015/16	4,622	4625
Rwanda	2019/20	1,666	1700
Tanzania	2022	3,730	3822
Uganda	2016	6,395	6326
Zambia	2018	6,130	6164
Zimbabwe	2015	4,917	5036
Total sample size		**56**,**163**	**56**,**657**

### Data source, and study population

For this study, the researchers specifically utilized the individual record (IR) files obtained from the surveys. Women and children are among the top targeted participants in DHS. The DHS is a survey conducted in more than 90 low- and middle-income countries, and it aims to gather data on crucial indicators including breastfeeding, fertility, family planning, immunization, HIV/AIDS, child health, and nutrition^23^. For this study, we included a total of 56,657 married reproductive-age women from 12 East African countries. These women had complete information on all the variables of interest. Malawi had the highest number of women included (4625), while Zimbabwe had the lowest number (5036) (see Table 1). When writing the manuscript, the researchers followed the ‘Strengthening the Reporting of Observational Studies in Epidemiology’ (STROBE) statement^24^. The dataset used in this study is available for free download at: https://dhsprogram.com/data/available-datasets.cfm.

### Sample size determination, and methods

DHS surveys are primarily conducted in developing countries, where statistical data may often be incomplete or outdated. Due to the outdated nature of sampling frames, which are statistical categories based on a national census, Macro follows a policy of employing a simple sampling design to ensure accurate implementation and control of fieldwork. As a result, most DHS surveys utilize a stratified, two-stage cluster sampling approach. This entails selecting primary sampling units (PSUs) in the first stage, with the probability of selection proportional to their size. In the second stage, a fixed number of households (or residential dwellings) is then chosen from a list of households obtained from the selected PSUs during an updating operation.

A PSU stands for Enumeration Area (EA), which is a specific area or part of an area. EAs are created based on the most recent population census and usually include multiple households. A complete list of EAs provides information on their geographical location, rural-urban characteristics, total population, and total number of households. Clear boundaries for these EAs are also defined in cartographic materials. However, as a population census is conducted only once every ten years, the information about EAs, such as the number of households, becomes outdated and needs updating. This updating process involves listing all households in the selected EAs and recording important information for each household, including the name of the household head, street address, and type of residence. Once this listing procedure is completed, a comprehensive list of households in the selected EAs is obtained, which serves as the sampling frame for the second-stage sampling used for household selection^25^. Following the listing of households, equal probability systematic sampling is used to select a specific number of households from inside the defined cluster^20^.

### Study variable measurements and definitions

#### Outcome variable

IPV consists of three forms of intimate violence, namely physical, psychological (emotional), and sexual violence. The last 12 months have seen women who are currently married experiencing emotional, physical, or sexual violence from their spouses. In the countries covered by DHS, women were surveyed using questions that began with ‘Does/did your husband/partner ever…’ If a woman answered yes to any of these questions, she was then asked about the frequency of the action in the last 12 months (whether it occurred often, sometimes, or not at all during that time).

#### Physical violence’s

To identify physical spousal violence, women were asked to confirm whether their husband pushed, shook, or threw something; slapped, twisted their arm, or pulled their hair; punched with his fist or with something that could hurt; kicked, dragged, or beat them up; tried to choke them; burned them; or threatened to attack with a knife, gun, or any other weapon within the last 12 months.

#### Sexual violence’s

To identify sexual spousal violence, women were asked to confirm whether their husbands physically forced them to have sexual intercourse even when they did not want to, physically forced them to perform any other sexual acts they did not want to, or forced them with threats or in any other way to perform sexual acts they did not want to.

#### Emotional/psychological violence’s

To identify emotional spousal violence, women were asked to confirm whether their husband says or does something to humiliate them in front of others, threaten to hurt or harm them or someone close to them, or insult them and make them feel bad about themselves.

#### Independent variables

Independent variables for the study were extracted from the DHS data. Socio-demographic characteristics of the mother (age of the woman, religion, region, educational level, occupation, residence, wealth index), sex of household head (headed by female, headed by male), husband’s education, husband’s working status, attitudes of women towards beating (negative, positive), cigarette smoking (yes, no), drinking alcohol (yes, no), currently pregnant, women’s decision-making autonomy (yes, no), and media exposure were computed as a composite variable that includes frequency of reading a newspaper or magazine, frequency of listening to radio, and frequency of watching TV. If a woman was exposed to at least one form of media in the last week, she would say “yes”; otherwise, she would say “no”. The wealth index was categorized into the poorest, poorer, middle, richer, and richest wealth quintiles according to the DHS standard. Total children ever born and fear of husband were also included in the study as variables that were identified as factors associated with intimate partner violence (IPV) after reviewing different literature.

### Data collection tools and procedure

Approximately every five years, the DHS office collects data that represents the nation. The focus is on the unique demographic and health challenges faced by each country. This is achieved through five different surveys, which include questionnaires for households, women, and men, as well as biomarkers and health institutions involving children. In this specific study, the individual questionnaire was utilized to gather information on the prevalence and domains of intimate partner violence (IPV), as well as the factors contributing to IPV among married women in East Africa. It is worth noting that this investigation adhered to the relevant statistical rules established by the DHS. To ensure comparability of data collected across different countries, the DHS program employs standardized methods and trains data collector professionals. This involves the use of uniform questionnaires, manuals, and field procedures.

### Data quality control

Before data collection, a pre-test was conducted in the DHS. Subsequently, a debriefing session was held with the field workers who were involved. Based on this session, any necessary adjustments to the questionnaires were made. Further information regarding the data collection process can be found in the DHS guidance, accessible in the Guide to DHS statistics.

### Statistical analysis

**Fig. 1:**
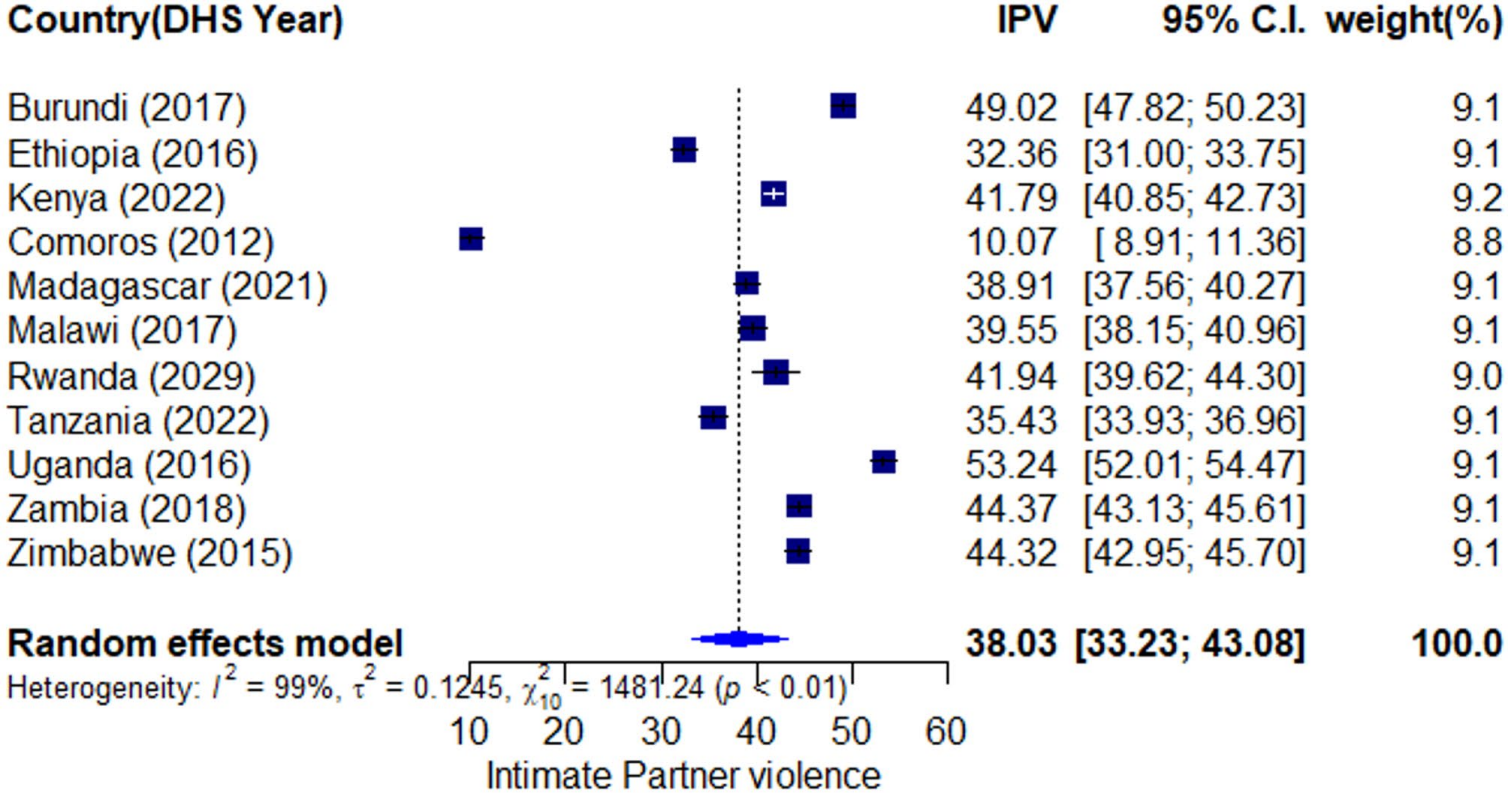
The pooled prevalence of IPV in East Africa (2012 to 2022).

The data were analyzed using Stata version 17.0, and Microsoft excel 2019. The analysis was conducted. To describe categorical variables, frequencies, and percentages were used. GSEM was used to determine factors associated with each domain of IPV, including physical, emotional, and sexual violence. Each domain of IPV was treated as a binary variable and analyzed using the binomial family with a logit link function. Husband-control behavior was considered a latent variable, consisting of items with yes/no responses. Its measurement model was analyzed using the binomial family with a logit link function. Statistical significance was determined using a P value < 0.05, with a confidence interval of 95%. Descriptive and multivariate analytical techniques were used to carve out the results. To identify significant factors associated with IPV among women in East African countries, a GSEM^26^ was performed with each form of IPV using a binary variable that was analyzed with the binomial family and a logit link function^27^. In the multivariate analysis, the partner’s controlling behavior was a latent variable (its values ranged from 0 to 6). The analysis started with a hypothesized model in Fig. 1. Modifications were taken iteratively by adding a path link. Finally, an over-identified model with minimum information criteria was retained. The final model was selected based on the statistical significance of the path coefficient, the theoretical meaningfulness of the relationship and minimum information criteria^27^. Results of the GSEM were reported as adjusted ORs (aOR) with their corresponding 95% CIs. All analyses were weighted to get unbiased estimates and carried out in STATA V.17.0 software (StataCorp, Texas, USA) using the ‘svyset’ command to adjust for the complex sampling structure of the data. Statistical significance was declared at *p* < 0.05.

### Generalized Structural Equation modeling development

Generalized Structural Equation Modeling (GSEM) is an extension of the Generalized Linear Model (GLM), originally introduced by Robert Nelder and Robert Wedderburn in the 1970s. Its development was fueled by advances in computing power and specialized software such as LISREL (Linear Structural Relations), pioneered by Karl Jöreskog and Dag Sörbom^28^.

**Fig. 2:**
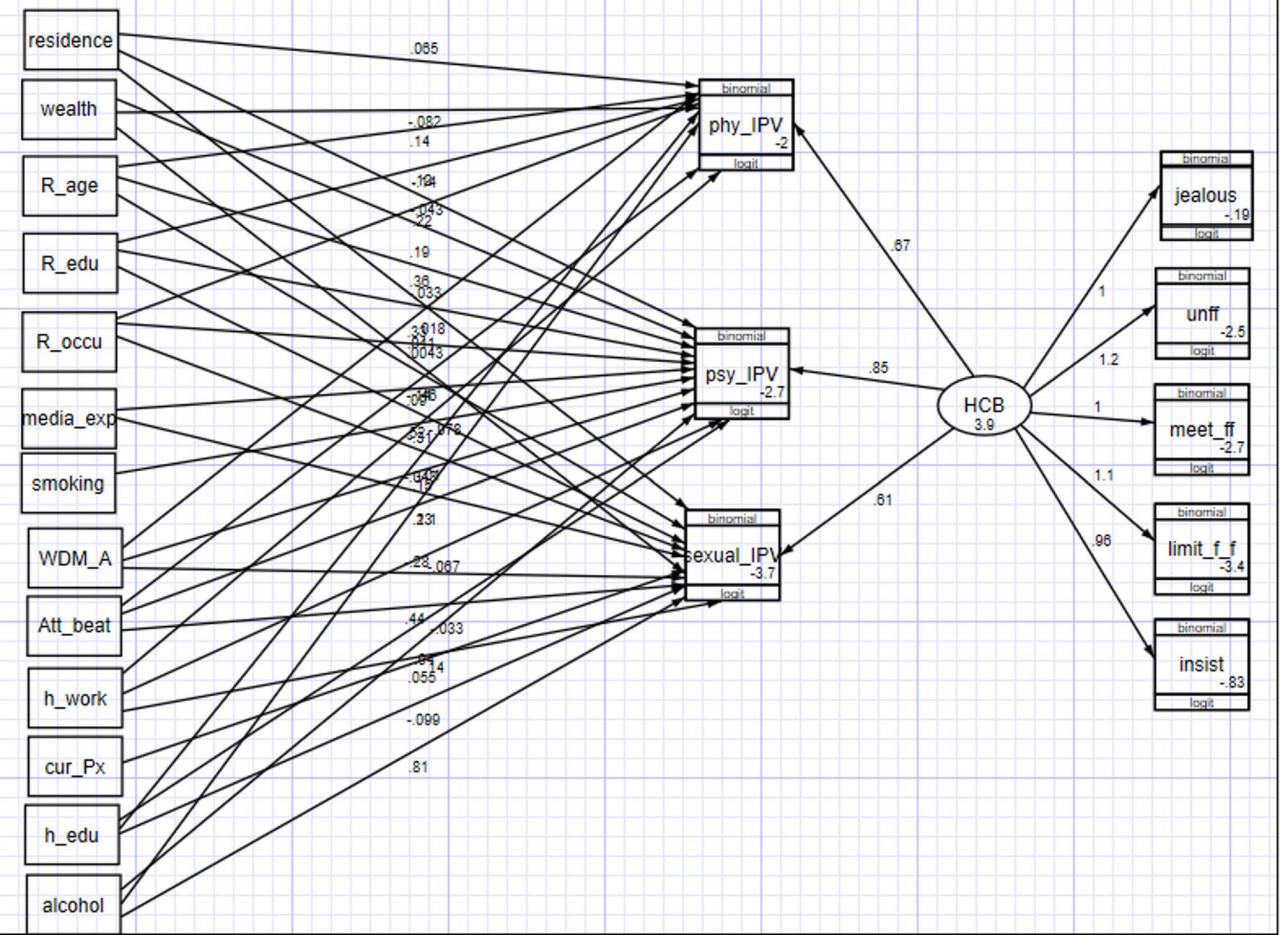
GSEM for associated factors of intimate partner violence among married reproductive age group women in Eastern Africa (2012 to 2022). phy_IPV = physical domain of intimate partner violence; psy_IPV = psychological domain of intimate partner violence; sexual_IPV = sexual domain of intimate partner violence; jealous = husband/partner jealous if respondent talks with other men; unff = husband/partner accuses respondent of unfaithfulness); meet_ff = husband/partner does not permit respondent to meet female frien; limit_f_f = husband/partner tries to limit respondent’s contact with family; insist = husband/partner insists on knowing where respondent is.

While structural equation modeling (SEM) was initially applied in the social and behavioral sciences, its scope has since expanded to public health, biological sciences, and medicine. GSEM combines elements of factor analysis and regression: it assumes a factor-analytic framework for measuring latent variables while simultaneously modeling relationships among latent and observed variables through regression structures. Latent variables are unobservable constructs that capture underlying dimensions (e.g., health-related quality of life, disease severity, or psychosocial factors). They are inferred through measurable observed indicators. **In** this study, for example, the latent construct of *husband controlling behavior (HCB)* was defined by six categorical indicators, such as restricting contact with friends or family and displaying suspicion of unfaithfulness (Fig. 2).

### Assumptions

GSEM extends the assumptions of GLM and traditional SEM, offering greater flexibility in handling clustered, longitudinal, and non-normally distributed data. This flexibility is particularly valuable in health research, where hierarchical structures and categorical outcomes are common. The core assumptions of GSEM include the recognition that observed variables are imperfect measures of latent constructs; latent constructs are modeled through factor analysis while their interrelationships are estimated using regression; the model accommodates both continuous and categorical observed variables; and dependencies such as spatial or hierarchical correlations can be incorporated into the framework. When these assumptions are not met, alternative modeling strategies may be applied. For example, generalized nonlinear models (GNMs) can be used to address highly skewed or non-normal outcomes such as binary or count data, while latent growth curve models are particularly useful for longitudinal data in which latent variables evolve over time, enabling researchers to capture growth trajectories and individual heterogeneity.

### Features of GSEM

Compared with conventional SEM, GSEM offers several additional features while omitting some SEM-specific ones. It allows generalized linear response functions, such as logit, probit, and Poisson, in addition to the linear functions used in SEM. It also accommodates multilevel models, which are not supported in standard SEM, and permits categorical latent variables, something SEM does not. Furthermore, GSEM supports factor-variable notation for model specification in software such as Stata, which enhances the efficiency and flexibility of model building. GSEM have many advantages compared to other models those strengths include^26^. GSEM’S ability to integrate models, handle diverse data, and accommodate complex structures makes it powerful tool for health researchers. It can give deeper insights into the relationships within health-related datasets.

## Results

### Characteristics of the study population

Data from 56,657 married reproductive age group women were included in the final analysis. Among these study participants, 24,663(42.53%) were between the Age of 25–34 years, and 41,890 (73.94%) of them were from rural part of the countries.About 39,798 (70.20%) were currently working. Nearly half (48.73%) of the respondents had no exposure to any mass media at all. Around 11.12% of the respondents were pregnant. Only 16,175 (28.55%) women had a decision-making autonomy, and 25,701 (45.36) women had an attitude towards wife-beating. Regarding the husband /parner of respondents, 24,293(43%) of them completed primary education, and 21,450(37.86%) of them drunk alcohol (Table 2).

**Table 2:** Socio-demographic characteristics of respondents in East African Countries from 2012 to 2022.

Variables	Frequency	Percentage
**Respondents age**		
15–24	13,593	23.99
25–34	24,663	43.53
35–49	18,401	0.22.48
**Residence**		
Urban	14,767	26.06
Rural	41,890	73.94
**Educational status**		
No education	11,702	20.65
Primary	25,961	45.82
Secondary/Higher	18,994	33.53
**Current working**		
No	16,859	29.76
Yes	39,798	70.24
**Media exposure**		
No	27,606	48.73
Yes	29,051	51.27
**Smoking Cigarettes**		
No	49,257	99.19
Yes	405	0.81
**Women’s decision making autonomy**		
No	40,479	71.45
Yes	16,175	28.55
**Attitude towards wife beating**		
No	30,955	54.64
Yes	25,701	45.36
**Current pregnancy**		
No or unsure	50,340.00	88.85
Yes	6,317.00	11.15
**Hausehold wealth status**	0.00	
Poorest	11,223.00	19.81
Poorer	11,223.00	19.81
Middle	11,051.00	19.51
Richer	11,923.00	21.04
Richest	11,237.00	19.83
**Partner’s/Husband’s age**		
15–19	295	0.52
20–24	4,011	7.08
25–29	9,510	16.79
30–34	11,354	20.04
35–39	10,600	18.71
40–44	8,090	14.28
45–49	5,835	10.3
50–54	3,526	6.22
≥ 55	3,434	6.06
**Partner’s/Husband’s educational status**		
No education	9,915	17.54
Primary	24,293	42.97
Secondary/Higher	22,326	39.49
**Partner’s/Husband’s current working**		
No	3,955	6.98
Yes	52,702	93.02
**Partner’s/Husband’s drink alcohol**		
No	35,207	62.14
Yes	21,450	37.86
**Total**	**56**,**657**	**100**

### Pooled prevalence of IPV in East Africa

The pooled prevalence of overall IPV across East African countries was 38.03% (95% CI: 33.23, 43.08). The highest prevalence was found in Uganda (53.24%), followed by Burundi (49%) and Zambia (44.37%). The lowest prevalence was reported in Comoros (10%). There was significant variation between countries, as indicated by I² = 99% (p < 0.01). Regarding the domains of IPV, the highest pooled prevalence was observed in the physical domain (26.15%, 95% CI: 22.56, 30.1) and the lowest in the sexual domain (11.6%, 95% CI: 8.44, 14.62). The psychological domain prevalence was 26.06% (95% CI: 22.83, 29.57). (Note: This likely references a separate forest plot Fig. 1). Figure 2 presents the path diagram of the Generalized Structural Equation Model (GSEM) used to identify factors associated with IPV. The model illustrates the hypothesized relationships between the observed variables (shown in rectangles) and the latent construct of ‘IPV’ (represented by the oval). The arrows represent direct pathways and their coefficients. As depicted, factors such as a partner’s jealousy (‘jealous’), accusations of unfaithfulness (‘unff’), restricting contact with friends (‘meet_ff’) and family (‘limit_f_f’), and insisting on knowing the respondent’s whereabouts (‘insist’) were all modelled as significant contributing factors directly loading onto the latent IPV construct. The model also shows the error terms for each observed variable.

### Factor associated with intimate partner violence in East Africa

As shown in the final model has included 14 exogenous variables of which one is latent variable (husband controlling behavior) and 13 are observed variables (women age, educational status, occupational status, household wealth index, residence, media exposure, smoking cigarettes, Current pregnancy, women decision making autonomy, Attitude towards wife beating, Partners educational status, partners working, and partners drink alcohol) and three endogenous (physical, emotional, and sexual violence) and five indicator variables that measure husband controlling behavior ( Fig. 2). Age, education, occupation, wealth index and husband controlling behavior were variables significantly associated with physical domain of IPV. Occupational status was significantly associated with psychological domain of IPV. Wealth index and husband controlling behavior were significantly associated with sexual domain of IPV (Table 3).

**Table 3:** Factors associated with intimate partner violence in East African countries (2012 to 2022).

Variables	Physical violence	Emotional violence	Sexual violence
	AOR (95% CI)	AOR (95% CI)	AOR (95% CI)
**Age**			
15–24	**Ref**	**Ref**	**Ref**
25–34	1.24 (1.17–1.31) *	1.29 (1.2–1.36)*	1.04 (0.97–1.12)
35–49	1.33 (1.23–1.41) *	1.49 (1.35–1.56)*	1.01(0.94–1.12)
**Residence**			
Urban	**Ref**	**Ref**	**Ref**
Rural	1.02 (0.96–1.09)	1.09 (0.991–1.17)	1.32 (1.21–1.43)*
**Educational status**			
No education	**Ref**	**Ref**	**Ref**
Primary	1.04 (0.98–1.11)	1.08 (1.00-1.14)*	1.04 (0.96–1.12)
Secondary/above	0.76 (0.7–0.82)*	0.94 (0.86, 1.03)	0.77 (0.69-0.85)*
**Household wealth**			
Poorest	**Ref**	**Ref**	**Ref**
Poorer	0.98 (0.92–1.05)	0.97 (0.90–1.05)	1.24 (1.13–1.35)*
Middle	0.9 (0.84–0.96)*	1.01 (0.94–1.10)	1.22 (1.10–1.32)*
Richer	0.9 (0.84–0.97)*	0.96 (0.88–1.04)	1.32 (1.17–1.42)*
Richest	0.72 (0.66–0.79)*	0.84 (0.76–0.93)*	1.20 (1.04–1.32)*
**Media exposure**			
No	-	**Ref**	**Ref**
Yes	-	1.10 (1.04–1.16)*	0.95 (0.89–1.013)
**Current working**			
No	**Ref**	**Ref**	**Ref**
Yes	1.2 (1.14–1.27)*	1.35 (1.27–1.43)*	1.63 (1.52–1.75)*
**Current pregnancy**			
No	-	-	**Ref**
Yes	-	-	1.06 (0.97–1.16)
**Smoking Cigarettes**			
No	-	**Ref**	-
Yes	-	0.74 (0.57–0.96)*	-
**Women’s decision-making autonomy**			
No	**Ref**	**Ref**	
Yes	1.02 (0.97–1.07)	1.17 (1.1–1.24)*	0.76 (0.71- 0 0.81)*
**Attitude towards wife beating**			
No	**Ref**	**Ref**	**Ref**
Yes	1.58 (1.52–1.66)*	1.25 ( 1.20–1.32)*	1.55 (1.46–1.64)*
**Husband’s educational status**			
No education	**Ref**	**Ref**	**Ref**
Primary	1.17 (1.1–1.25)*	1.17 (1.09–1.26)*	1.06 (0.97–1.145)
Secondary/Higher	0.99 (0.92–1.05)	0.97 (0.89–1.05)	0.84 (0.76–0.92)*
**Husband current working**			
**No**	**Ref**	**Ref**	**Ref**
**Yes**	0.91 (0.84–0.99)*	0. 93 (0. 84-1.03)	1.12 (0.99–1.26)
**Husband’s drink alcohol**			
No	**Ref**	**Ref**	**Ref**
Yes	3.06 (2.92–3.2)*	2.55 (2.43–2.68)*	2.24 (2.12–2.37)*
HCB	1.92 (1.87–1.97)*	2.32 (2.25–2.4)*	1.82 (1.78–1.87)*

**NB: HCB** = husband controlling behavior; **AOR**: adjusted odds ratio; **CI**: confidence interval: * = 5% segnficancy; **Ref** = Reference.

Given other variables are constant, women aged 25–34 and 35–49 exhibit 24% and 33% higher odds of physical violence compared to those aged 15–24, (AOR:1.24, 95% CI: 1.17–1.31) and (AOR:1.33 ,95% CI: 1.23–1.41) respectively. Educational attainment plays a role, where women with secondary education or above are 24% less likely to experience physical violence than those with no education (AOR: 0.76, 95% CI: 0.7–0.82).

Household wealth also appears influential, keeping other variables constant, women from richest households experiencing 28% lower odds of physical violence (AOR: 0.72, 95% CI: 0.66–0.79). Being employed increases the likelihood of experiencing physical violence by 20% (AOR: 1.2, 95% CI: 1.14–1.27). Additionally, women’s who had attitudes towards wife beating showing 58% higher odds of experiencing physical violence (AOR: 1.58, 95% CI: 1.52–1.66), given other variables are constant keeping other variable constant, women whose husband with primary education and those currently not working are associated with 17% increased and 10% decreased odds of physical violence (AOR: 1.17, 95% CI: 1.1–1.25) and (AOR: 0.91, 95% CI: 0.84–0.99) respectively. Furthermore, women whose husband/partner drinks alcohol experiences 3.06 times higher odds of physical violence compared to those their husband did not drink alcohol (AOR: 3.06, 95% CI: 2.92–3.2).

Given other variables are constant, women aged 25–34 and 35–49 exhibited 29% and 49% higher odds of emotional violence compared to those aged 15–24(AOR: 1.29 ,95% CI: 1.2–1.36) and (AOR:1.49, 95% CI: 1.35–1.56) respectively. A Women who completed primary education experiences 8% higher odds of emotional violence (AOR: 1.08, 95% CI: 1.00-1.14), compared to with no education. Keeping other variables constant, women from richest household wealth category exhibited 16% lower odds emotional/Psychological violence (AOR: 0.84, 95% CI: 0.76–0.93) compared to those from poorest household wealth category. Media exposure is linked to increased odds of emotional violence by 10% (AOR: 1.10, 95% CI: 1.04–1.16). Being employed is associated with higher odds of emotional violence by 35% (AOR: 1.35, 95% CI: 1.27–1.43). Additionally, women’s who had decision-making autonomy (AOR: 1.17, 95% CI: 1.1–1.24) and attitudes towards wife beating (AOR: 1.25, 95% CI: 1.20–1.32) were both associated with increased odds of emotional violence.

Regarding to, husbands’ characteristics, the odds of experiencing emotional violence was 2.55 time higher among women whose partner/husband drinks alcohol (AOR: 2.55, 95% CI: 2.43–2.68) compared to their counter parts. Coming to sexual violence part, keeping other variables constant, women residing in rural areas experienced 32% higher odds of sexual violence compared to those in urban areas (AOR:1.32, 95% CI: 1.21–1.43). Moreover, women from poorer to richest households exhibit increased odds of sexual violence compared to the poorest, with the highest odds observed among those in the richest category (AOR: 1.20, 95% CI: 1.04–1.32). Additionally, being employed significantly increases the likelihood of experiencing sexual violence by 63% (AOR: 1.63, 95% CI: 1.52–1.75). Women’s decision-making autonomy appears to serve as a protective factor against sexual violence, with those possessing greater autonomy exhibiting 24% lower odds (AOR: 0.76, 95% CI: 0.71–0.81) compared to their counter parts. Women’s who had attitudes towards wife beating exhibiting 55% higher odds of sexual violence (AOR: 1.55, 95% CI: 1.46–1.64) compared to those women’s who had no attitudes towards wife beating. Keeping other variable constant, women whose husband with secondary/higher education were associated with 16% decreased odds of sexual violence (AOR: 0.84 (0.76, 0.92) compared to their counter parts. Furthermore, women whose husband/partner drinks alcohol experienced 2.24 times higher odds of emotional violence compared to those their husband did not drink alcohol (AOR:2.24, 95% CI: 2.12, 2.37).

## Discussion

The current study identified key factors associated with different forms of IPV (physical, emotional, and sexual) in East African countries. The findings revealed that sociodemographic, behavioral, and attitudinal characteristics of women and their partners were significant factors of IPV.

According to this study, age was significantly associated with IPV, women aged 25–34 and 35–49 experience significantly higher odds of physical and emotional violence compared to younger women aged 15–24. For physical violence, the adjusted odds ratios (AORs) were 1.24, and 1.33 more times, respectively. For emotional violence, the AORs were 1.29 and 1.49 more times respectively. This aligns with a report done by WHO in 2018^29^ and prior researches done among pregnant women in East Africa^30^, among adolescents in East Africa^31^, implying that increased exposure to abusive partners and other changes in relationship dynamics may cause IPV risk to rise with age. However, the association between age and sexual violence is not significant, reflecting complex trends that may warrant further investigation.

Based on the finding of this study rural women were more likely to experience sexual violence 1.32 times more than urban women. This aligns with findings done on women in East Africa^30,32^, that rural areas often have more traditional norms that tolerate or support IPV. This may be explained by women who do want assistance but struggle to get it because of their geographical isolation, lack of transportation, and inability to access their income^33,34^. Higher educational attainment in women correlates with reduced IPV exposure. Secondary or higher education significantly reduces physical and sexual violence. This aligns with previous studies done in United States^35^, Vietnam^36^, and Nepal^37^, suggesting that education enhances self-empowerment and fosters awareness of rights, enabling women to challenge abusive behavior and educated women might have better resources and negotiation power within relationships^38^. However, primary education is associated with a slightly increased risk of emotional violence, suggesting that higher educational levels may be necessary to observe robust protective effects against this form of violence.

The study reveals wealthier households show a protective effect against physical and emotional IPV but not sexual IPV. For instance, the richest category significantly lowers the odds of physical violence, and emotional violence respectively. This aligns with previous studies done in Vietnam^36^, and Philippines^39^. The persistence of sexual violence across wealth categories may reflect power dynamics unaffected by economic status^40^. Given that spouses may react negatively to financial empowerment, this paradox could be a reflection of conflict and control dynamics in partnerships^30,32^. Women currently working face increased risks of all three types of IPV, with the highest risk for sexual violence. This is in line with a study done in Philippines^39^.This might reflect male partners’ backlash against women’s economic independence^41^.

The current study at hand reveals that, smoking cigarettes was significantly associated with emotional violence, women who smoked were 26% less likely to experience emotional violence. This finding is unexpected as smoking is often linked to increased aggression or stress-induced behaviors. It runs counter to a study that was published in Nicotine & Tobacco Research and shows that smoking prevalence is higher among people who have experienced emotional violence because of the negative effects of stress and the absence of other coping mechanisms^42^. However, this reduction might reflect complex sociocultural factors or coping mechanisms among smokers that mitigate certain types of violent expressions^43,44^. Further research could explore whether smokers’ lower emotional violence rates stem from altered interpersonal dynamics, economic considerations, or other contextual factors. Women who justify wife beating have consistently higher odds of all IPV types. The odds of physical violence, emotional violence, and sexual violence increase by 58%, 25%, and 55% respectively. It is similar to a studies done in Vietnam^36^ and Uganda, showed that individuals justifying wife-beating were significantly more likely to experience or perpetrate emotional, physical, and sexual IPV^45,46^. These points to deep-rooted patriarchal norms that perpetuate violence as a socially acceptable practice^45,47–50^.

While decision-making autonomy correlates positively with emotional violence (AOR: 1.17), and it reduces the odds of sexual violence (AOR: 0.76). This dual effect may indicate that attempts for empowerment have occasionally encountered opposition while also offering protection from specific types of abuse^51^. According to a supportive study conducted in Ghana and Ethiopia, women who shared decision-making authority in the home with their spouses were much less likely to be victims of physical, emotional, or sexual intimate partner violence^52,53^. However, IPV was more common among women who had very little or very much autonomy, suggesting that balancing decision-making is crucial to reducing the likelihood of violence^52,53^. Similarly, media exposure appears to increase the odds of emotional violence (AOR: 1.10), possibly reflecting greater awareness or reporting among media-literate women^54^. It is similar with a research done in Sub-Saharan Africa shown that women exposed to media have slightly higher odds of reporting emotional IPV^52^. According to their explanation, media exposure may increase awareness and reporting of IPV rather than directly influencing its occurrence^52^.

According to the findings of this study, husbands’ alcohol consumption is the strongest predictor of IPV across all forms. For physical violence, emotional violence and sexual violence, the odds increase threefold (AOR: 3.06), greater than twofold (AOR: 2.55), and (AOR: 2.24) greater than twofold respectively. It is supported by meta-analysis studies in Sub-Saharan Africa, which has consistently found that alcohol consumption by husbands or male partners is one of the strongest predictors of all forms of IPV^52^. Similar results from research conducted throughout East Africa ascribe this to the cognitive and behavioral impacts of alcohol, which worsen aggression attitudinal and empowerment factors and impair self-control^30,32^.​ Husband’s current working status was inversely associated with physical violence. Women whose husbands employed were 9% less likely to experience physical violence compared to those with unemployed husbands (AOR: 0.91). The reduced likelihood of physical violence among women whose husbands employed may reflect decreased financial stress or increased stability in households where the husband is working^55,56^. However, the lack of significant associations for emotional and sexual violence indicates that other sociocultural or personal factors might play a more dominant role in these forms of IPV. Some of the observed associations were counterintuitive, such as higher media exposure and employment being linked to increased odds of IPV, and smoking appearing to reduce emotional IPV. These findings should be interpreted with caution, as the mechanisms underlying these relationships remain unclear. Additionally, the cross-sectional nature of the study precludes establishing causality; the associations reported represent correlations rather than direct cause-effect relationships.

### Theoretical and practical implications

The findings of this study contribute to the growing body of literature on violence by providing context-specific evidence that enhances theoretical understanding of how violence manifests and is experienced within the studied population. From a theoretical perspective, the results highlight the complex interaction between individual, social, and structural determinants of violence. These findings support existing theoretical frameworks that conceptualize violence as a multifactorial phenomenon shaped by socio-cultural norms, power relations, and institutional contexts.

From a practical perspective, the study provides important insights that may inform policy development and intervention strategies aimed at preventing violence and mitigating its consequences. The evidence generated can assist policymakers, public health professionals, and community stakeholders in designing targeted prevention programs and strengthening support systems for affected individuals. Furthermore, the findings underscore the importance of integrating violence prevention initiatives into broader public health and social protection strategies. By translating research evidence into actionable recommendations, the study contributes to efforts aimed at reducing violence and promoting safer and healthier communities.

## Conclusions

The pooled prevalence of IPV among East African women was notably high. The highest prevalence of overall IPV found in Uganda. The lowest prevalence of overall IPV was reported in Comoros. The study analyzed factors associated with intimate partner violence (IPV) in East African countries from 2012 to 2022, focusing on physical, emotional, and sexual violence. Key findings indicate that older age groups are significantly more likely to experience physical and emotional violence compared to the young age group. Rural residence is significantly associated with higher odds of sexual violence. Educational status, household wealth, media exposure, current working status, women’s decision-making autonomy, attitude towards wife beating, husband’s educational status, and husband’s alcohol consumption are all significant factors influencing various forms of IPV.

### Recommendations

To effectively reduce IPV and improve the safety and well-being of women in East Africa, targeted interventions should be implemented for women, who are at higher risk of physical and emotional violence. Additionally, strengthening interventions in rural areas is crucial to addressing the higher incidence of sexual violence in these regions. Promoting education for both women and men, with a particular focus on improving secondary and higher education access for women, is essential as higher educational attainment is associated with reduced IPV. Economic empowerment initiatives for women, especially in the poorest and middle wealth quintiles, can also mitigate IPV risk. Utilizing media exposure to disseminate information on IPV prevention and women’s rights is important due to its significant impact on reducing emotional violence. Furthermore, introducing workplace policies and support systems for working women can address the increased odds of IPV associated with employment. Lastly, implementing community programs aimed at reducing alcohol consumption among husbands is vital, given its significant correlation with all forms of IPV. By addressing these interconnected areas, policymakers and stakeholders can develop more effective strategies to combat IPV in East Africa.

### Implications of the study

#### Policy and intervention development

Findings from SEM studies can inform the development of evidence-based policies and interventions to address IPV among women in Africa. By identifying the key factors associated with IPV and understanding their interrelationships, policymakers and practitioners can design targeted interventions that address the root causes and risk factors specific to this population. This can include initiatives focused on economic empowerment, gender equality, community mobilization, and mental health support.

#### Contextualized understanding

SEM studies provide a more nuanced and context-specific understanding of IPV among women in Africa. Factors influencing IPV can vary across different regions, cultures, and communities. By conducting SEM in a specific rural context, researchers can uncover the unique risk and protective factors that contribute to IPV in that setting. This localized understanding can guide the development of culturally sensitive interventions and prevention strategies.

#### Informing future research

SEM studies on IPV among women in Africa can generate hypotheses and identify gaps in knowledge. The findings can guide future research by highlighting areas that require further investigation or replication in different populations. This can contribute to a growing body of literature on IPV in Africa and inform the development of more comprehensive theoretical frameworks to explain and address IPV dynamics. In summary, studying IPV among women in Africa using SEM offers a robust analytical approach to understand the complex dynamics and underlying factors associated with IPV. This research can have important implications for policy development, intervention design, and future research efforts aimed at preventing and addressing IPV in African communities.

## Data Availability

The datasets used and/or analysed during the current study available from the corresponding author on reasonable request.
